# A second-generation computational modeling of cardiac electrophysiology: response of action potential to ionic concentration changes and metabolic inhibition

**DOI:** 10.1186/1742-4682-11-46

**Published:** 2014-10-21

**Authors:** Nour Eddine Alaa, Hamid Lefraich, Imane El Malki

**Affiliations:** Department of Mathematics, Laboratory of Applied Mathematics and Computer Science (LAMAI), Faculty of Science and Technology, Cadi Ayaad University, Abdelkarim Elkhattabi Avenue, Marrakech, Morocco

**Keywords:** Cardiac action potential, Cable equation, Reaction-diffusion system, Electro-migration, Nonlinear coupled system, Finite element method, Nernst-Planck equation, Numerical analysis, Substrate suicide, Computational simulation

## Abstract

**Background:**

Cardiac arrhythmias are becoming one of the major health care problem in the world, causing numerous serious disease conditions including stroke and sudden cardiac death. Furthermore, cardiac arrhythmias are intimately related to the signaling ability of cardiac cells, and are caused by signaling defects. Consequently, modeling the electrical activity of the heart, and the complex signaling models that subtend dangerous arrhythmias such as tachycardia and fibrillation, necessitates a quantitative model of action potential (AP) propagation. Yet, many electrophysiological models, which accurately reproduce dynamical characteristic of the action potential in cells, have been introduced. However, these models are very complex and are very time consuming computationally. Consequently, a large amount of research is consecrated to design models with less computational complexity.

**Results:**

This paper is presenting a new model for analyzing the propagation of ionic concentrations and electrical potential in space and time. In this model, the transport of ions is governed by Nernst-Planck flux equation (NP), and the electrical interaction of the species is described by a new cable equation. These set of equations form a system of coupled partial nonlinear differential equations that is solved numerically. In the first we describe the mathematical model. To realize the numerical simulation of our model, we proceed by a finite element discretization and then we choose an appropriate resolution algorithm.

**Conclusions:**

We give numerical simulations obtained for different input scenarios in the case of suicide substrate reaction which were compared to those obtained in literature. These input scenarios have been chosen so as to provide an intuitive understanding of dynamics of the model. By accessing time and space domains, it is shown that interpreting the electrical potential of cell membrane at steady state is incorrect. This model is general and applies to ions of any charge in space and time domains. The results obtained show a complete agreement with literature findings and also with the physical interpretation of the phenomenon. Furthermore, various numerical experiments are presented to confirm the accuracy, efficiency and stability of the proposed method. In particular, we show that the scheme is second-order accurate in space.

## Background

Cardiac disease is the leading cause of deaths worldwide. A proportion of them is caused by rhythm irregularities of the heart, such as atrial fibrillation. In the healthy heart, the cardiac contraction is produced by softly propagating non-linear electrical waves of excitation. Any disturbance in conduction or coordination of electrical signals can result in abnormal heart rhythms, so called arrhythmias. Bradycardia, tachycardia, heart block, and atrial and ventricular fibrillation are examples of arrhythmias. The stimulation of cardiac cells is instigated by a sudden change in the electrical potential across the cell membrane due to the transmembrane flux of charged ions. The release and propagation of an electrical signal, which is ensured by controlled opening and closing of ions channels, is one of the most important functions of the cell. About fifty-two years ago, the first continuous mathematical model of cardiac cell designed to reproduce cell membrane action potentials is presented by Hodgkin and Huxley [1]. Ever since, many complex models have been developed for cardiac cells inspired by their approach. Most of these models can be classified in three sets. 1) “First generation” of ionic models which are able to reproduce basic ionic currents such as the Beeler-Ruter (BR) [2] and Luo-Rudy-I (LR-I) [3] models. 2) “Second generation” of models, which in addition to a biophysically detailed description of ion channel, pump and exchanger currents, also contain the intracellular ionic concentrations such as the DiFrancesco-Noble [4]. 3) simplified models that only contain the minimum set of phenomenological currents necessary to reproduce mesoescopic features of cell dynamics, e.g., conduction velocity (CV) restitution and action potential (AP) restitution [5, 6]. Generally, simulations based on first and second generation models are computationally demanding, however it is often desirable to designate the minimum key characteristics necessary to characterize a specific phenomenon and then proceed by using simplified models.

The purpose of this paper is to design a new computer model of cardiac action potential, which can be classified in the set of second generation models as in addition to a detailed description of ion channels, it also includes detailed description of intracellular concentrations. However, given the trend of researchers in the field, which aims to offer models with less computational complexity, we made a further simplification in the ions representation, the so called mean-field approximation of ionic solution, in which ions are not considered as microscopic discrete entities but as continuous charge densities. This leads us to a fully continuous model, Nernst-planck equations for the species concentrations and a modified cable equation for the electrical potential. This model has the advantage of besides containing a detailed biophysical description of the cardiac activity is less computationally demanding because of the simplification we made. This model is more general than those in literature of membrane transport as it extends them in three topics: 1) it’s a multidimensional model, 2) it allow accessing both time and space domains for the transport equation and also for the electrical potential equation, 3) it includes different reaction kinetics terms.

## Introduction

In this paper we consider a class of models of cardiac cell signaling, which includes ions migration through biological membranes. The electrical propagation is described by a new cable equation by assuming that the membrane acts as an electrical circuit in which resistance and capacitance are arranged in a parallel circuit. The incorporated migrations exist for most living cells and some biochemical processes, where the motion of ions is supposed due to diffusion and to the effect of the electrical field. Furthermore ions undergo biochemical reactions. So the ions concentrations satisfy the Nernst-Planck equations, including a kinetic reaction terms and the potential is given by a new cable equation. The model is given by:
1

where *Q*_*T*_=]0,*T*[×*Ω*, , *T*>0; *Ω* is an open regular set of  which represents the biological cell and *∂Ω* represents the cell membrane. *Ω* is supposed time independent because we are not interested in cell volume control, so we neglected variations of cell size. For each *i*, *C*_*i*_ is the concentration of the *A*_*i*_ species which has diffusion coefficient *d*_*i*_, mobility *m*_*i*_ and valency *z*_*i*_. *ϕ* is the electrical potential, *D* is a positive diffusion coefficient, *F*_*a*_ is Faraday constant, *δ*_(0,0)_ is a dirac at the point (0,0) which represents the center of the cell, *C*_*m*_ is the membrane capacitance, *ϕ*_*rest*_ is the resting potential, and *F*_*i*_ are reaction terms. For each *i*, *F*_*i*_ denotes the production rate of the species *A*_*i*_ due to all homogeneous reactions in which it is involved. We suppose that *F*_*i*_ depends continuously on the *C*_*j*_’s, and that *d*_*i*_ is a positive constant for each *i*.
2

In this paper we present a numerical simulation of such systems, for a large class of reaction kinetics, however, for the applications we considered a suicide substrate reaction. This article is organized in the following way. The next section is devoted to the modeling of the problem. Then, we did a finite element discretization of the mathematical model. After that we present applications, results and numerical experiments showing the accuracy, efficiency and stability of the proposed method. Finally, conclusions are drawn in the last section.

## Governing equations

### Modeling the electromigration of ions

Let us consider a cardiac cell which fills the bounded open set *Ω* of , *N*≥ 1. This type of reaction within the membrane always contain electroactive ions  as one of their major components. The movement of ions is supposed to be due to diffusion and also the effect of electrical field. The mass conservation equation for the species *A*_*i*_ is
3

where *C*_*i*_ is the concentration of species *A*_*i*_, *F*_*i*_ denotes the production rate of *A*_*i*_ due to all the homogeneous reactions in which it is involved and *J*_*i*_ is its molar transport flux. Let’s mention that the concentration *C*_*i*_ can also change because of the production or destruction of the species *A*_*i*_ within the cell, which justify the production rate *F*_*i*_ that represents the net rate of increase of *A*_*i*_ (production-destruction). When *F*_*i*_ is positive, the cell is a source (leading to an increase in the total amount of *A*_*i*_), and when *F*_*i*_ is negative, it’s a sink. The functions *F*_*i*_ are called also source functions. Furthermore, migration is included along with diffusion as possible modes of transport of each *A*_*i*_. The molar flux *J*_*i*_ then becomes
4

There is a relationship between the mobility *m*_*i*_ and the diffusion constant *d*_*i*_ (see [7]), which is given by
5

where *z*_*i*_*F*_*a*_ is the charge carried by a mole of species *A*_*i*_, *R* is the universal gas constant and *T*_*e*_ is the local temperature. The transport equation of each *A*_*i*_ becomes
6

### Modeling the electrical potential

The cell membrane can be modeled as a capacitor related in parallel with variables resistances and batteries. The capacitance is due to the phospholipid bilayer that separates the ions on the inside and the outside of the cell. The resistances and batteries represent the different ionic currents. Consequently, the electrophysiological behavior of a single cell is governed by the following differential equation [1]


where *ϕ* is the voltage, *t* is the time, *I*_*ion*_ represents the sum of the ionic currents flowing across the cell membrane, *I*_*stim*_ is an applied external stimulation current, and *C*_*m*_ is cell capacitance per unit surface area. Analogously, a 2D single cardiac cell can be modeled as a continuous system with the following partial differential equation [1]


where *ρ*_*x*_ and *ρ*_*y*_ are respectively the cellular resistivity in the *x* and *y* directions, *S*_*x*_ and *S*_*y*_ are respectively the surface-to-volume ratio in the *x* and *y* directions, and *I*_*ion*_ represents the total ionic current. In 2D, our purpose wasn’t to study the effects of anisotropy, consequently we take *ρ*=*ρ*_*x*_=*ρ*_*y*_ and similarly *S*=*S*_*x*_=*S*_*y*_. By considering a “diffusion” coefficient *D*=1/(*ρSC*_*m*_), it follows


In the present model the ionic current depends on all ions existing in the cell, then


by specifying that the stimulation current is applied at the center of the cell (0,0), it follows that


## Variational formulation of the problem

In order to show the numerical formulation of the problem, let *V*=*L*^2^(0,*T*;*H*^1^(*Ω*)) be the space of approximate solutions and *W*=*H*^1^(*Ω*) be the space of tests functions. Let *W*^*h*^ be a finite element space of Lagrange *P*1 included in *W* and *V*^*h*^=*L*^2^ (0,*T*;*W*^*h*^) be the finite dimensional subspace of *V*. The Faedo-Galerkin formulation for the problem is given by, finding *C*_*i*,*h*_∈*V*_*h*_ for *i*=1,…,*N*_*s*_ and *ϕ*_*h*_∈*V*_*h*_ such that *ϕ*_*h*_=0 in *∂Ω*:


where  are the projections of *C*_*i*,0_,*ϕ*_0_ on *W*_*h*_.

According to the boundary conditions we have


∀*w*_*h*_∈*W*_*h*_, for 1≤*i*≤*N*_*s*_, we define the function


For all *v*_*h*_∈*W*_*h*_ such that *v*_*h*_=0 in *∂Ω*, we define the function


The weak formulation of the system becomes, finding *C*_*i*,*h*_∈*V*_*h*_ for *i*=1,…,*N*_*s*_ and *ϕ*_*h*_∈*V*_*h*_ such that *ϕ*_*h*_=0 in *∂Ω*:
7

## Channel blockers in the treatment of cardiac arrhythmias

Channel blockers (CB’s) are a type of drugs which binds to the enzyme inside the pore of a specific type of ion channel and blocks the flux of ions through it. Channel blockers are useful agents in antiarrhythmic drug therapy, especially supraventricular tachyarrhythmias [8–10]. Furthermore, there is many genetic diseases that modify and block cardiac ion channels, causing cardiac channelopathies [11]. Consequently, to model such a channel inhibition, we need to establish the transport system for suicide substrate reaction.

### Suicide substrate kinetics

An enzyme system of major experimental concern; see [12, 13], is the mechanism-based inhibitor, or suicide substrate system, represented by Walsh et al. [14],
8

where *E*, *S* and *P* stand for enzyme, substrate, and product, respectively; *X* and *Y*, enzyme-substrate intermediates; *E*_*i*_, inactivated enzyme; and the *k*’s are positive rate constants.

In this system, *Y* has a choice of one of two pathways, namely, to *E*+*P* with rate *k*_3_ or to *E*_*i*_ with rate *k*_4_. The ratio of these rates, *k*_3_/*k*_4_, is called the partition ratio and is denoted by *r*. Each of these pathways are supposed to be irreversible over the timescale of the reaction see [15]. *S* is known as a suicide substrate because it binds to the active site of an enzyme—like a substrate—but the enzyme converts it into an inhibitor which irreversibly inactivates the enzyme. Thereby, the enzyme ‘commits suicide’. In this way, a suicide substrate can specifically target an enzyme for inactivation. Furthermore, suicide substrates are particularly useful in drug administration, as they are not noxious in their common form and only the designated enzyme can convert them to their inhibitor form. For example, suicide substrates have been subject of investigation for use in the treatment of depression (monoamine oxidase inhibitors, Seiler et al. [12]), epilepsy (brain GABA transaminase inhibitors, Walsh [13]), and some tumors (ornithine decarboxylase inhibitors, Seiler et al. [12]). Suicide substrate kinetics have been studied by Waley [15] and by Tatsunami et al. [16], who had interest in the factor which determined whether the substrate was exhausted before all the enzyme was inactivated. Waley proposed it was *rμ*, where *μ* is the ratio of the initial concentration of enzyme to that of substrate, namely, *e*_0_/*s*_0_. Tatsunami et al., on the other hand, found the determining factor to be (1+*r*)*μ*. When (1+*r*)*μ*>1 the substrate is exhausted, while for (1+*r*)*μ*<1, all the enzyme is inactivated. When (1+*r*)*μ*=1, both occur. The interest is when *e*_0_/*s*_0_ is not small, which was in effect assumed since both Waley [15] and Tatsunami et al. [16] used a quasi-steady state approximation. The validity decreases for increasing values of *e*_0_/*s*_0_. We denote the concentrations of the reactants by


The law of mass action applied to (8) leads to one equation for each reactant and hence a system of nonlinear equations. We obtain the following system
9

## Numerical scheme

In this section, we present the numerical scheme for solving the problem, we used for the time marching scheme an implicit scheme for the transport equations and an explicit second order Runge-Kutta [17] scheme for the potential equation.

### Time marching scheme

Our method is based on an explicit second order Runge-Kutta scheme for the potential equation and an implicit scheme for the transport equations. To this end, let us denote by  and  the approximate value at time *t*=*t*^*n*+1^ and *t*=*t*^*n*^, respectively and by *δt* the time step size. Then by using (7) and the following algorithm, we determine the unknown fields.

#### Algorithm of resolution

We used the following algorithm to calculate *ϕ*_*h*_ and *C*_*i*,*h*_.

- Initialize for *i*=1,…,6
- Loop over *n*

At step *n*:

- Calculate  solution of

where:


and


where:

- Calculate  solutions of:

initialize  for *i*=1,…,6,

Loop over *k* untill


## Results and discussion

In this section, aiming to understand how an action potential emerges from the mathematical structure that we have developed we study the dynamics of the model for different types of input. Pulse input, time-dependent input, and sinusoidally varying amplitude input are considered in turn. These input scenarios have been chosen so as to provide an intuitive understanding of dynamics of the model. We present the behavior of the Voltage in response to a short current input, a time dependent input, and a sinusoidal current input. To describe signaling in a cell body, this one can be assumed to be an ellipse. For all the results of this section, we considered the following parameters:

For the computations cell capacitance per unit surface area is taken as *C*_*m*_=2.0 *μF*/*cm*^2^ and surface to volume ratio is set to *S*=0.2 *μm*^-1^, following Bernus et al. [18]. Taggart et al. [19] found the velocity for conductance along the fiber direction in human myocardium 70 *cm*/*s*, which required a cellular resistivity *ρ*=162*Ωcm* for Tusscher et al. [20]. This is of the same magnitude of *ρ*=180*Ωcm* used by Bernus et al. [18] and the *ρ*=181*Ωcm* used by Jongsma and Wilders [21], and it leads to a diffusion coefficient *D*=1/(*ρSC*_*m*_) of 0.00154 *cm*^2^/*ms*. The cell is represented by an ellipse with semi-major axis a = 2 and semi-minor axis b = 1. The diffusion coefficients of the ions are *d*_1_=10^-3^*m*^2^.*s*^-1^, *d*_2_=2.10^-3^*m*^2^.*s*^-1^, *d*_3_=5.10^-3^*m*^2^.*s*^-1^, *d*_4_=10^-3^*m*^2^.*s*^-1^, *d*_5_=2.10^-3^*m*^2^.*s*^-1^, *d*_6_=4.10^-6^*m*^2^.*s*^-1^, the reaction parameters are *k*_1_=2 *s*^-1^, *k*_-1_=4 *s*^-1^, *k*_2_=12 *s*^-1^, *k*_3_=10 *s*^-1^ and *k*_4_=2 *s*^-1^. The charge number of the ions are *z*_1_=1, *z*_2_=-1,*z*_3_=1, *z*_4_=1, *z*_5_=1 and *z*_6_=-1. The initial concentrations are *e*_0_=0.5 *μM* and *s*_0_=0.5 *μM*;*ϕ*_*rest*_=0 and *ϕ*_0_=-80 *mV*. The data employed for the reaction parameters and initial concentrations were taken from Burke et al. [22]. The time step of the simulation is *dt*=10^-5^*s*, and *T*=0.006 *s*. The stimulus current *I*_*stim*_, is the key to stimulate the system. In the heart, the excitation is ensured by the Sino-Atrial Node. Here we applied a single stimulus, which delivers a short current pulse of 1 *ms* and strength -200 *μA*/*cm*^2^, beginning at *t*=120×10^-5^*s* at the center of the cell. Let’s mention that the Dirac at (0.0) can be approached by *f*= exp(-1000(*x*^2^+*y*^2^)).

### Numerical result 1: electrophysiological validation of the model

To validate the model three criterion are considered 1) excitability 2) All or none 3) action potential morphology.

#### Excitability

**Figure 1:**
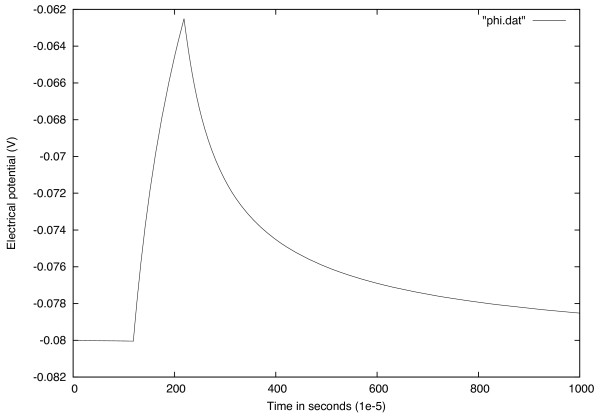
**Generated AP by stimulus current at time** ***t*** **=120** ***.*** **10** ^**-5**^ ***s*** **.** Before time *t*=120.10^-5^ *s* no stimulation is applied.

This means that the cell is in its resting potential as long as no stimulation is applied to it. However, by using an efficient stimulus it produces AP. Figure 1 shows that before time *t*=120.10^-5^*s* there was no stimulation and consequently there is no action potential but at time *t*=120.10^-5^*s* an AP is generated.

#### All or none

**Figure 2:**
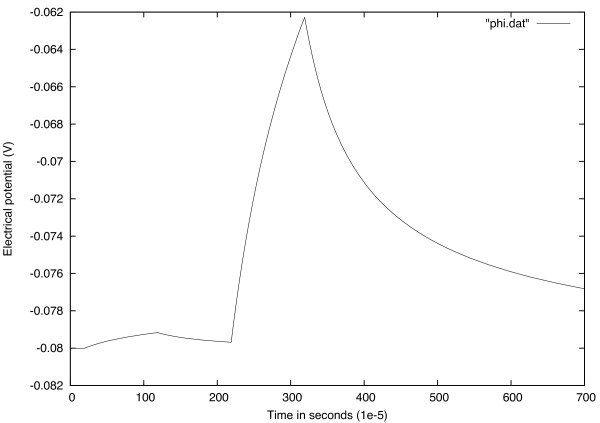
**If the stimulus current is smaller than threshold mount, AP does not generate.**

This means that if the amplitude of the stimulus pulse is equivalent to the threshold, an action potential is generated, but if it’s lesser than threshold AP is not generated. Figure 2 shows using a stimulus current which is less than threshold AP does not generate.

#### Action potential morphology

This model has characteristics of cardiac cell as it reproduces the triangular AP morphology (see Figure 1) with no sustained plateaus, which is similar to the AP shape obtained with more complex models Nygren et al. [23] and cherry et al. [24]. Furthermore, the figure is similar to the action potential block by saxitoxin in the book [25].

### Numerical result 2: response to a time dependent current input

**Figure 3:**
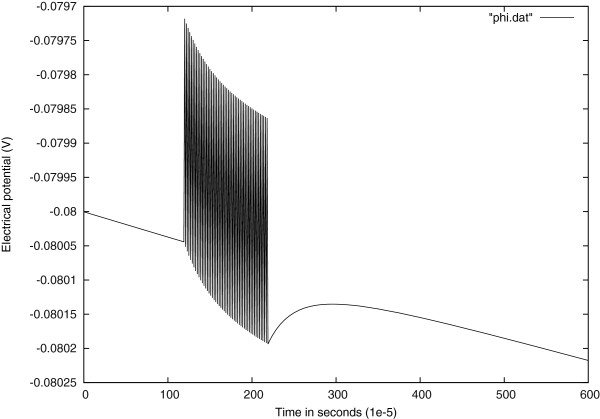
**Voltage response to a time dependent current input at the center of the cell.**

In order to explore a more realistic input scenario, we stimulate the model by a time dependant input current  of 1*ms* duration, starting at *t*=120×10^-5^*s* at the center of the cell. It can be observed that the stimulus change of sign at each time step, the results are shown in Figure 3.

**Figure 4:**
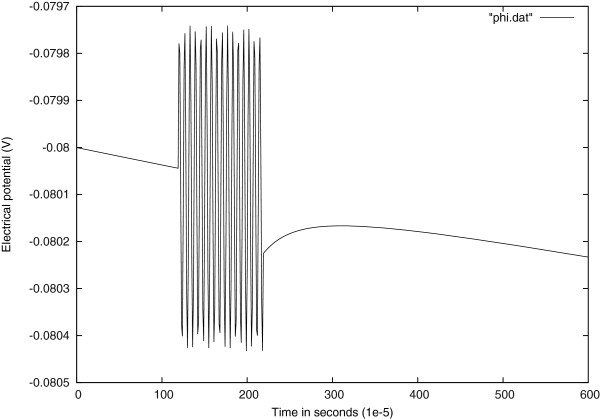
**Voltage response to current input at the center of the cell with a sinusoidally varying amplitude.**

Figure 4 shows the response of electrical potential to a stimulus with a sinusoidally varying amplitude.

### Numerical result 3: visualizing current spread and its impact on the product and substrate concentrations

**Figure 5:**
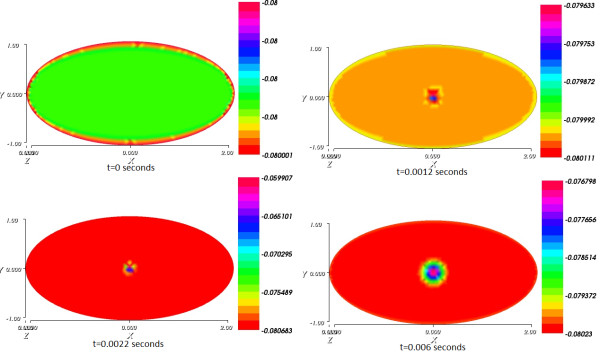
**The propagation of an electrical signal through the cell from initial time t = 0 to final time T = 0.006 s.**

Figure 5 plots the spread of potential through the cell. The system is stimulated by the same stimulus defined in the beginning of the section.

**Figure 6:**
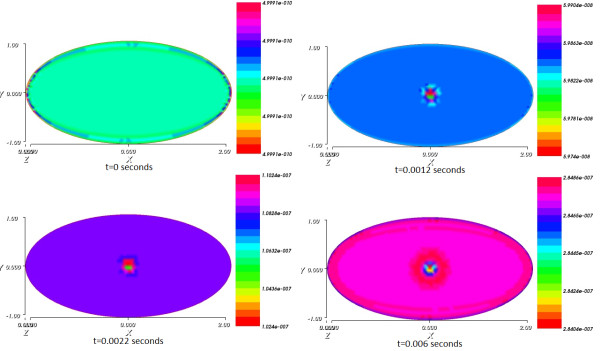
**Spatial distribution of the product concentration from initial time t = 0 to final time T = 0.006 s.**

Figure 6 shows the impact of the current spread on the product concentration as it presents the spatial distribution of the product concentration from the initial time to final time.

**Figure 7:**
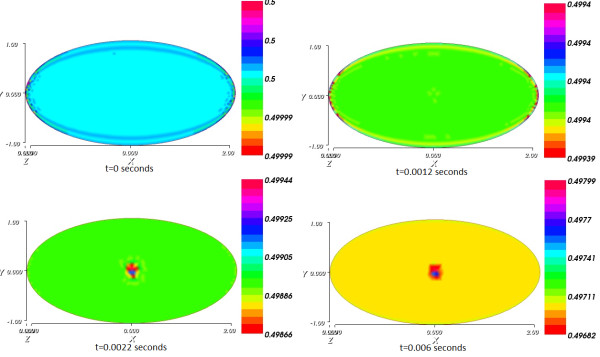
**Spatial distribution of the substrate concentration from initial time t = 0 to final time T = 0.006 s.**

Figure 7 shows the impact of the current spread on the substrate concentration as it presents the spatial distribution of the substrate concentration from initial time to final time.

## The convergence test

The rate of convergence of the scheme is difficult to prove analytically. However, numerical experimentation suggests that the scheme is second-order accurate in space. A quantitative estimate of the convergence error was obtained by performing a number of simulations for the same initial condition on a set of increasingly finer space meshes and time steps. The initial conditions are constants. Let *T*_*h*_ the mesh generation of *Ω*, and *h*(*T*_*h*_)=*max*{*diam*(*e*_*k*_)|*e*_*k*_∈*T*_*h*_}, we take *h*=0.1,*h*=0.15 and *h*=0.2. For each mesh we integrate to time *T* with . Note that as we refine the space step we also refine the time step. The error of the numerical solution was defined as .

In Table 1 is presented the error of convergence for different mesh sizes.

**Table 1:** **Error of convergence for different mesh sizes**

Mesh size	h1 =0.2	h2 =0.15	h3 =0.1
Error	19.10^-5^	14.10^-5^	95.10^-6^

## Stability and accuracy tests

**Figure 8:**
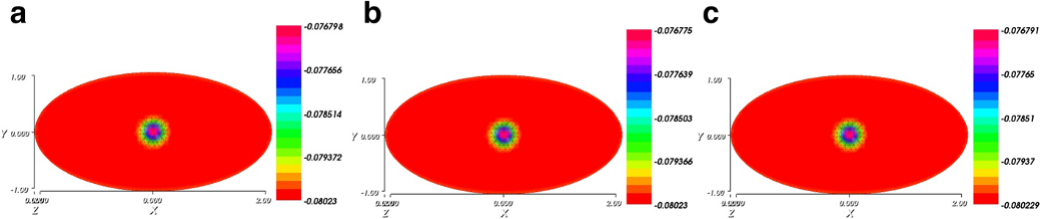
**Snapshots of the electrical potential at T = 0.006 s with three different time steps.** The time steps are shown below each figure. **(a)** Time step dt = 0.00001 **(b)** Time step dt = 0.000005 **(c)** Time step dt = 0.0000025. Legend: values of concentrations through the cell.

Now, let us give some information about the numerical stability of our algorithm. We perform a numerical experiment with different time step *dt*,  and . These results suggest that the scheme is indeed stable as the solutions are quasi the same for different time steps. To illustrate, we chose to represent the electric potential, and the product concentration. In Figure 8, we display snapshots of the electrical potential at time T = 0.006 s with three different time steps dt = 0.000005, dt = 0.0000025 and dt = 0.00001. We can see that the results are quasi the same at the final time T.

**Figure 9:**
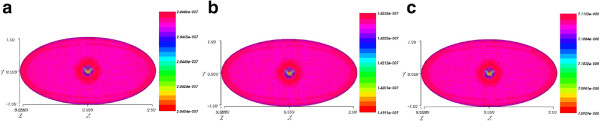
**Snapshots of the product concentration at T = 0.006 s with three different time steps.** The time steps are shown below each figure. **(a)** Time step dt = 0.00001 **(b)** Time step dt = 0.000005 **(c)** Time step dt = 0.0000025. Legend: values of concentrations through the cell.

In Figure 9, we display snapshots of the product concentration at time T = 0.006 s with three different time steps dt = 0.0000025, dt = 0.000005, and dt = 0.00001. We can see that the results are quasi the same at the final time T.

**Figure 10:**
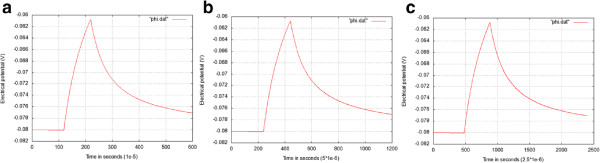
**The evolution of the electrical potential for three different time steps.** The time steps are shown below each figure. **(a)** Time step dt = 0.00001 **(b)** Time step dt = 0.000005 **(c)** Time step dt = 0.0000025.

In Figure 10, we present the evolution of the electrical potential at the center of the cell for three different time steps dt = 0.0000025, dt = 0.000005, and dt = 0.00001. We can see that the results are quasi the same.

## Conclusion

In this paper, a new second-generation model of the set simulating cardiac action potential is proposed by using a more general mathematical model and a numerical technique based on the finite element method. The electrophysiological validation of the problem shows that the model has all characteristics of cardiac cells: excitability, all or none, and the triangular AP morphology which is similar to those obtained with more complex models Nygren et al. [23] and Cherry et al. [24]. Moreover, after comparison we can observe a complete consistency with literature findings [25]. A variety of numerical experiments were presented to confirm the accuracy, efficiency, and stability of the proposed method.
